# A nationwide time-series analysis for short-term effects of ambient temperature on violent crime in South Korea

**DOI:** 10.1038/s41598-024-53547-6

**Published:** 2024-02-08

**Authors:** Seulkee Heo, Hayon Michelle Choi, Jong-Tae Lee, Michelle L. Bell

**Affiliations:** 1 School of the Environment, Yale University, 195 Prospect St, New Haven, CT 06511 USA; 2 Interdisciplinary Program in Precision Public Health, Department of Public Health Sciences, Graduate School of Korea University, Seoul, South Korea

**Keywords:** Environmental sciences, Environmental social sciences

## Abstract

Psychological theories on heat-aggression relationship have existed for decades and recent models suggest climate change will increase violence through varying pathways. Although observational studies have examined the impact of temperature on violent crime, the evidence for associations is primarily limited to coarse temporal resolution of weather and crime (e.g., yearly/monthly) and results from a few Western communities, warranting studies based on higher temporal resolution data of modern systemic crime statistics for various regions. This observational study examined short-term temperature impacts on violent crime using national crime data for the warm months (Jun.–Sep.) across South Korea (2016–2020). Distributed lag non-linear models assessed relative risks (RRs) of daily violent crime counts at the 70th, 90th, and 99th summer temperature percentiles compared to the reference temperature (10th percentile), with adjustments for long-term trends, seasonality, weather, and air pollution. Results indicate potentially non-linear relationships between daily summer temperature (lag0–lag10) and violent crime counts. Violent crimes consistently increased from the lowest temperature and showed the highest risk at the 70th temperature (~ 28.0 °C). The RR at the 70th and 90th percentiles of daily mean temperature (lag0–lag10), compared to the reference, was 1.11 (95% CI 1.09, 1.15) and 1.04 (95% CI 1.01, 1.07), indicating significant associations. Stratified analysis showed significant increases in assault and domestic violence for increases in temperature. The lagged effects, the influences of heat on subsequent crime incidence, did not persist 21 days after the exposure, possibly due to the displacement phenomenon. We found curvilinear exposure–response relationships, which provide empirical evidence to support the psychological theories for heat and violence. Lower public safety through increased violent crime may be an additional public health harm of climate change.

## Introduction

Crime has been recognized as a public health problem since at least the mid-1980s^1^ as crime and public health issues have similar origins of social and environmental factors, such as deprivation and low social capital^2,3^. Violence is an important destroyer of human well-being and quality of life^4,5^. In particular, fear of violent crime is a major cause of mental health issues such as anxiety^6^. Violence is a complex outcome emerging from a complicated causal system with multiple risk factors^5^. Climate change and extreme weather events are environmental factors that have profound effects on human behaviors, socioeconomic systems, security, and social stabilization^7,8^. Climate change can directly or indirectly influence the behaviors of individuals or groups which can lead to crime. However, the number of observational studies on climate change and crime is yet notably lower compared to the literature on climate change and its impacts on mortality or disease incidence, and many unanswered questions remain.

Numerous studies have discussed associations between temperature, aggression, and violence for nearly four decades. According to the well-known routine activity theory, warm temperatures have an indirect link with violence as people change their routine activities and warmer conditions bring potential victims and offenders in closer proximity resulting in increases in violent crimes^4,9,10^. This is a supporting theory for the increases in violent crimes at warm conditions compared to cold conditions^11^. There have been studies assuming that heat stimulates aggression and violence through increased discomfort, frustration, and impulsivity since 1970s^12^. Several psychological heat-aggression theories exist for heat and aggression, which were reviewed thoroughly by Anderson^13,14^. For example, the “simple negative affect model” assumes that aggressive behaviors occur at temperature extremes (e.g., extremely cold, extremely hot) due to the consequence of hostility and aggression led by uncomfortable feelings. In contrast, the “negative affect escape model” assumes that aggressive behaviors will be the highest in the moderate temperature ranges and decrease at the temperature extremes due to the escaping tendency (i.e., the tendency to leave a thermodynamic state by either a physical or a chemical process) becomes stronger. In addition, laboratory studies proposed a hypothesis that thinking of heat as opposed to cold or neutral thoughts can be likely tied to having aggressive thoughts, feelings, and behaviors^15^. There also exists evidence for the pathogenic impacts of high temperature on aggression and impulsivity through the activation of both the part of the brain responsible for thermoregulation and emotion regulation^15^. The pathways include neuroinflammation in the brain, central nervous system functions, imbalance of serotonin, neurotoxicity, production of extra adrenaline caused by an acute failure of thermoregulation and a hyperthermia^16–18^.

Climate-related risk factors would likely increase the burdens of violent crimes^19,20^. A new mechanism theory of the climate change aggression model suggests two pathways for climate change-induced increases in violence^21,22^. First, the increases in frequency and integration of heat could directly affect irritability in individuals leading to aggressive thoughts. Second, increases in natural disasters (e.g., hot weather events, drought, flood) could decrease agricultural production, shift the economy to poverty and resource competition, and increase intergroup or civil wars, conflicts, and terrorism^20,23–25^. Another new model called the Climate, Aggression, and Self-control in Humans (CLASH) model explores heterogeneity within and between countries in the aggression and violence associations^24,26^. It assumes that people will adapt to changing seasonal variability in different ways depending on regions (e.g., latitudes) and national wealth^8^. For instance, people at higher latitudes, where the climate is too harsh to grow crops, could develop long-term future-oriented planning self-control for survival. The lack of self-control is a plausible mediator for linking the effects of temperature on aggression and violence. Therefore, the CLASH model assumes that temperature and its seasonal variation are an important situational factor in shaping culture, such as how individuals and groups develop social tools to avoid aggression and violence. A large amount of evidence for the putative mechanisms between high temperature, aggression, and violence indicates the need for more research attention on the associations between heat and violent crime and sustainable decision-making on climate change and public safety, but the study results for this topic are yet limited^11^.

The associations between heat and violence have been well established by modern research with crime statistics and temperature data. A few analytical frameworks have been applied in these population-based observational studies to examine the associations between temperature and violent crime. Research foci varied including (1) identifying correlations between temperature and violent crimes, (2) identifying the seasonality of violent crimes^4,27^, and (3) examining the temperature-violent crime relationship functions using the modern crime data at the monthly or annual scales^19,19,23,28,29^. The potential correlations between temperature and violent crimes were examined in historical studies by comparing the conflict rates among geographical regions with different climates or through a long time period (e.g., decades) with temperature anomalies for a population^13,18^. A few studies (in the US) have suggested a small but significant positive correlation between long-term (e.g., annual) high ambient temperature and violent crimes. For example, Anderson et al. reported that the violent crime rate was higher in hotter years in the 50 largest US cities^30^. Later studies focused on rapid changes in violent crime in response to short-term changes in temperature at shorter time scales (e.g., hourly to yearly scales). Monthly variations in violent crimes with higher rates in hotter seasons (e.g., Summer) across various regions, including several US cities, European cities, and Beijing, China^4,31–33^, indirectly implied that violent crimes and higher temperature may be associated.

As an example of the third framework (i.e., examining the temperature-violent crime relationship functions), four US studies conducted in Chicago, Illinois; Boston, Massachusetts; Philadelphia, Pennsylvania: and Dallas, Texas suggested curvilinear relationships (e.g., an inverted U-shaped curve) between daily temperature and violent crime. A study using data from 2997 US counties showed that temperature has positive curvilinear effects on criminal behaviors at monthly scales^34^. A recent study in Japan examined the short-term effects of high temperatures on violent crime, focusing on the time series of temperature and daily violent crimes with adjustments for the seasonality of violent crimes in the statistical model^35^. However, evidence for associations between the daily variation in temperature and violent crime is largely limited to a few US cities and uncertainly remains for other populations and regions. Furthermore, most previous studies^35^ using temperature data with varying temporal resolution, from monthly to daily, mainly focused on increases in violent crimes at the longer term high temperature (e.g., temperature during the warm months) compared to the cold temperature (e.g., temperature during the cold months). Given the strong seasonality of the violent crimes within a year shown in numerous studies, significant positive associations between temperature and violent crime seem to be apparent. It is, however, unclear if short-term changes in temperature during the summer periods are associated with rapid changes in violent crimes. This is an important topic as the number of extremely hot days in addition to the average temperature are expected to increase under climate change, posing burdens for public health and social safety. Furthermore, evidence is limited for the dynamics of violent crime incidence during hot days regarding the duration of the effects of heat on crime. The number of heat-related events (e.g., violent crime) generally increase immediately after the high exposure (e.g., on the same of or several days after exposure). There is knowledge gap for the effects of heat on events well after an exposure (i.e., lagged effects)^36^, for heat and incidence of violent crime, which is an important consideration in accurate examinations of the influence of heat on violent crime. More studies are needed to examine heat-violence associations with the best available data and econometric methods, focusing on understudied geographical regions and modeling varying lagged effects. The current time-series analysis focused on analyzing the associations between summer temperatures and daily counts of violent crimes using official statistics of crime compiled by police agencies in South Korea.

## Materials and methods

### Study population

South Korea is in a temperate climate zone and has distinct four seasons of spring, summer, fall, and winter. The summer is hot and humid while the winter is cold and dry. South Korea is divided into several different administrative units. The administrative units “Metropolitan Si” and “Do” are the primary ones (roughly analogous to US counties). The average size of these level-1 areas is 5930 km^2^. Within these geographical units, the second-level unit (hereafter “level-2 administrative areas”) is “Si”, “Gu”, or “Gun”, and the third-level unit is “Eup”, “Myeon”, or “Dong.” We collected weather and crime data for the level-2 administrative areas and the unit of analysis is the day of each level-2 administrative area. The number and boundary of administrative areas in South Korea occasionally change as some areas merge into a new administrative region or an administrative region is divided into multiple divisions. Some areas were within the jurisdiction of police offices in other administrative areas until a new police office was introduced. As the daily crime statistics in these areas did not exist prior to the new police offices, they were excluded from our analysis for the consistency of the data. As a result, we included 201 level-2 administrative areas in our analysis, while there were 261 level-2 administrative areas, for example, in 2022. The average size of the level-2 administrative area is 399.5 km^2^ (roughly analogous to US cities) (minimum 2.8 km^2^, maximum 1816.2 km^2^) (Supplementary Fig. S1 online). Most previous studies of temperature and health outcomes such as mortality used the level-1 administrative units to assess exposure–response relationships. In the current study, we used the smaller level-2 administrative areas to better estimate exposure levels for the study areas with a spatially higher resolution and consider heterogeneity across the study regions.

### Data

The police-recorded crime data for 1 January 2016 through 31 December 2020 were obtained from the Smart Policing Big Data Platform^37^. The crime statistics are generated based on the Law for Complication and Management of Police Criminal Statistics. According to this law, police officials in charge of criminal investigations should prepare crime incidence statistics when they become aware of a crime through accusation, report, or recognition. Crime records are reported by each police station and then compiled by the Korean National Police Agency. The Smart Policing Big Data Platform, which provides customized big data through collaboration between the police, the public, and the private sectors, provided the dataset of high precision for daily crime incidence data for various types of crime for this research. In generating statistics, the Smart Policing Bigdata Platform used 13 crime categories: murder, robbery, burglary, assault, sexual crime, domestic violence, fraud, public indecency, juvenile crime, voice phishing, misdemeanor, traffic offense, and other crime. Some crime categories have sub-categories. For example, sexual crime includes three sub-codes: sexual assault, dating violence, and stalking. Juvenile crime includes two sub-codes: school violence and juvenile delinquency. However, the data we obtained for this research included the primary 13 crime categories and not the subcategories. The dataset included information on the date of crime, the name and address (e.g., level-2 administrative unit) of the police office, the number of police officers for a given police office, the number of total crimes, and the number of incidence for each category of crime per day for a given police office. To assess the associations between high temperature and violent crime, we defined violent crimes as murder, robbery, assault, or domestic violence. Sexual crime and juvenile crime were not included due to the sub-categories of behaviors that do not necessarily include violence (e.g., juvenile delinquency). As this study focused on interpersonal violent crime, we did not examine intrapersonal or inward violence such as suicide^38^.

We matched each police office to its administrative areas (i.e., jurisdiction) using the police station name and address information, and then the number of crimes was aggregated for each administrative area, day, and type of crime. In the crime dataset, there were 252 police offices, one for level-2 administrative area in this study. A total of 4,753,113 violent crimes across the entire country for the 201 level-2 administrative areas in our study (23,647 violent crimes for each area on average) were included in the analysis for the study period (i.e., 2016–2020).

The National Meteorological Administration provided the daily measurements of ambient temperature (e.g., mean, maximum, minimum) and dew point temperature for each monitoring station across the nation between 1 January 2016 and 31 December 2020. The weather monitoring data from 626 monitoring stations in total were aggregated for each day and each of the 201 level-2 administrative areas.

Hourly air quality monitoring data for PM_2.5_ and O_3_ were obtained from the Korea Environment Corporation^39^ including 496 monitoring stations across the country. The daily values of air pollutants for each study area were calculated using hourly observations from all monitoring stations within each level-2 administrative area. The correlations among temperature metrics and air pollution concentrations are shown in the Supplementary File (Tables S1 and S2 online).

#### Statistical analysis

The outcome variable of this study is daily violent crime counts. Given the different temperature ranges across the study areas, this study used percentile values of temperature representing the temperature distribution of each study area (i.e., level-2 administrative unit) in assessing the associations between temperature and violent crime. We limited our analysis to the summer months (e.g., Jun–Sep) and therefore our analysis represents changes in daily temperature during the summer months.

We assessed how short-term exposure to high temperature is associated with changes in daily violent crime counts using distributed lag non-linear models (DLNM) within a quasi-Poisson family^40,41^. The DLNMs are commonly used in environmental studies for over-dispersed outcomes. The DLNMs can consider complex lagged dependencies through a cross-basis function describing the combination of two functions of the exposure–response relationship and the additional lag-response relationship^41,42^. Also, DLNMs allow a non-linear exposure–response function that is more closely aligned with some psychological theories suggesting that the impact of heat on aggression is non-linear. Our study applied a single DLNM for each target region and this approach has a strength that it can identify the region-specific threshold temperature and relationship curves accounting for the heterogeneous temperature ranges, population’s adaptation level to local climate, and any unobserved contextual determinants for exposure–response relationships^43,44^.

The overall statistical analysis was conducted in two stages. In the first stage, we applied DLNMs separately for each study area to assess estimates of area-specific associations between temperature and violent crime counts. For each area, we also considered the daily mean temperature and daily maximum temperature as temperature metrics in separate models. In order to estimate non-linear relationships, we used a quadratic B-spline for the area-specific temperature percentile with internal knots at the 50th and 90th temperature percentiles with two boundary knots and a natural cubic spline for lags with an intercept and two internal knots placed at equally spaced values in the log scales. Previous studies^45,46^ have applied varying lag periods for the temperature effects on health outcomes (e.g., mortality), for example, 25 days. We examined the lagged effects of the temperature for up to 10 days (e.g., day 0—day 10) and up to 21 days (day 0–day 21)^47^. The models for each area included a natural cubic B-spline for calendar time (degrees of freedom [df] = 3) and daily mean dew point temperature (df = 3). The seasonality of the outcome variable was adjusted by applying a natural cubic B-spine of day of the season with equally spaced knots and 4 degrees of freedom (i.e., df = 4). The models also included categorical variables for holidays and day of the week as potential confounders. The degrees of freedom for each parameter were chosen based on the quasi-Akaike information criterion (Q-AIC) value across the study areas^48^.

In the second stage, we applied a random-effects meta-analysis with the restricted maximum likelihood (REML) estimator to derive the pooled overall cumulative associations between temperature and violent crime counts. Exposure–response models require a reference value of exposure for estimating the associations so that the results can be interpreted as the effect of the exposure versus a reference^48^. For this, the basis functions were centered on a reference temperature (e.g., 10th percentile) of each temperature metric (i.e., daily mean temperature, daily maximum temperature). The choice of the reference value depends on interpretational purposes and does not affect the fit of the exposure–response relationships^48^. Using the area-specific estimates from the first stage, we estimated pooled risk ratios (RRs) and respective 95% confidence intervals (CIs) at the 70th, 90th, and 99th percentile temperatures compared to the reference temperature. These percentile values (i.e., 70th, 90th, and 99th) were selected to represent moderate, hot, and extremely hot temperatures during the summer season.

Our main focus was the daily counts of violent crimes (i.e., murder, robbery, assault, domestic violence). However, we examined how the high temperature was associated with each sub-category of violent crime and if the associations differ among them. We stratified analysis for the sub-categories of violent crimes, namely, assault and domestic violence. The models did not converge for murder or robbery as an outcome potentially due to the small sample size.

As an additional measure of summer heat, we applied additional DLNM models including the categorical variable of heat wave for each study day. The same set of variables in the main DLNMs was also included. Using the conventional definitions of heat waves by the Korea Meteorological Administration, we defined heat wave days in two ways: days with the daily maximum temperature ≥ 33 °C for two or more consecutive days^49^; and days with the daily maximum temperature ≥ 35 °C for two or more consecutive days. The impact of heat waves was assessed as RRs with 95% CIs (i.e., risks on heat wave days vs. risks on non-heat wave days).

We conducted some sensitivity analysis. First, we limited the main analyses to the years 2016–2019 to exclude the potential influences of the COVID-19 pandemic and to the year 2020 to include only the COIVD-19 pandemic. Second, we examined the temperature-violent crime associations for all of 2016–2020, rather than the subset of warm months only. Third, we assessed the cumulative RRs through lag0–lag5 instead of lag0–lag10 or lag0–lag21. Fourth, we conducted the DLNMs with additional adjustments for daily PM_2.5_ and O_3_ concentrations for 2016–2020.

To examine the robustness of non-linear associations between exposure and response in different statistical models, we examined the associations between summer temperature and daily counts of crime, using Poisson Pseudo Maximum Likelihood regression (PPML) with multiple-levels of fixed-effects as a sensitivity analysis. PPML models can be applied to the count measurements with a significant amount of observations of zero values and any number of fixed-effects^50^. The use of Poisson regressions with high-dimensional fixed effects can be used for several analytic purposes including the control of heterogeneity across different populations and over time^51,52^. The estimated results are robust to other estimators such as ordinary least squares estimators (OSL). In these Poisson regressions, we used the daily crime counts of each level-2 administrative area as a dependent variable. As applied in our DLNMs, we used the area-specific percentiles of the daily mean temperature through lag0–lag10 as the main exposure variable. To consider non-linear relationships between temperature exposure and crime counts, we divided the lag0–lag10 temperature converted into the percentiles into six same-length intervals (0–16th, 17–33rd, 34–50th, 51–67th, 68–84th, 85–99th percentiles). The exposure–response relationship is linear within a bin. Each day of a given administrative area was assigned a value of one for *i*-th dummy variable when the percentile of lag0–lag10 temperature falls in *i*-th temperature bin. The same set of confounders as our DLNMs was included in these models (i.e., day of the week, holidays, and daily dew point temperature). The models included fixed effects at the administrative region with respect to year and month to account for unobserved heterogeneity both across regions and over time and cluster standard errors at the level of administrative areas. The Poisson regressions were applied to each type of violent and non-violent crime. The command for PPML can show very sensitivity numerical problems that the algorithms do not converge or convergence is achieved by overfitting the zeros of counts, and fail on statistical software^53^. Even though a convergence is achieved, obtained estimates can be spurious^53^. Due to this numerical problem, results were not shown for counts of murder and robbery that had a very large number of zero values.

Crime incidence may be underreported depending on the type of crime. For example, a study suggested that domestic violence may be reported relatively immediately, whereas there may be a delay between the occurrence and reporting of sexual assault. Even for the same type of crime, it is not clear if the reporting rate is the same dependent on temperature or if different reporting rates on hotter days would bias the associations between temperature and crime incidence on hotter days. We note that our data for daily crime incidence is the best available data for the crime statistics with a high temporal resolution (i.e., daily), but our analyses were not able to quantify the degree of under- or overreporting of crime, including potential variation by type of crime.

The “dlnm” and “mvmeta” packages of the R statistical program (version 3.5.1) were used for the analysis.

## Results

Table 1 shows the descriptive statistics of the population, counts of crime, and environmental variables. During the summer months, there was a total of 13,195,173 counts of crime, of which 1,525,851 were for violent crimes. The percentage of violent crimes among all crimes was 11.6% in the warm seasons (Jun–Sep) and 11.8% overall (i.e., across all seasons). The average of daily violent crimes for each area was 13 counts during the study period.

**Table 1:** Descriptive statistics of the variables for the study populations (2016–2020).

	All seasons (jan–dec)	Warm seasons (jun–sep)
Annual population (10^5^ persons)		
2016	5261.8	–
2017	5267.3	–
2018	5276.0	–
2019	5278.2	–
2020	5262.0	–
Crime		
Total crime (N, %)	35,171,457 (100)	13,195,173 (100)
Violent crime (N, %)	4,146,474 (11.8)	1,525,851 (11.6)
Murder (N, %)	4082 (0.01)	1532 (0.01)
Robbery (N, %)	3852 (0.01)	1350 (0.01)
Assault (N, %)	3035,721 (8.6)	1,122,620 (8.5)
Domestic violence (N, %)	1,102,819 (3.1)	400,349 (3.0)
Burglary (N, %)	1,126,010 (3.2)	414,067 (3.1)
Sexual crime (N, %)	293,457 (0.8)	118,612 (0.9)
Juvenile crime (N, %)	529,692 (1.5)	221,755 (1.7)
Fraud (N, %)	227,006 (0.6)	75,037 (0.6)
Public indecency (N, %)	702,082 (2.0)	248,353 (1.9)
Voice phishing (N, %)	163,347 (0.5)	59,661 (0.5)
Misdemeanor (N, %)	14,127,264 (40.2)	5,529,123 (41.9)
Traffic offense (N, %)	7,840,862 (22.3)	2,803,464 (21.2)
Other crime (N, %)	6,015,263 (17.1)	2,199,250 (16.7)
Temperature		
Daily mean temperature (°C) (mean, SD)	13.2 (9.7)	23.5 (3.4)
Daily maximum temperature (°C) (mean, SD)	18.7 (9.8)	28.5 (3.9)
Daily minimum temperature (°C) (mean, SD)	8.4 (10.3)	19.6 (3.9)
Daily mean dew point temperature (°C) (mean, SD)	6.6 (11.6)	18.9 (4.2)
Air pollution		
Daily mean PM_2.5_ (µg/m^3^) (mean, SD)	22.6 (14.1)	17.1 (10.0)
Daily mean O_3_ (ppm) (mean, SD)	0.029 (0.013)	0.032 (0.013)

The averages of the area-specific 10th, 70th, 90th, and 99th percentiles daily mean temperature during the summer months were 19.3 °C, 25.4 °C, 28.0 °C, and 29.9 °C, respectively. The corresponding values for the daily maximum temperature were 23.8 °C, 30.4 °C, 33.5 °C, and 36.2 °C.

The counts of violent crime stratified by year, level-1 administrative area, and type of crimes are shown in Supplementary Figs. S2–S4. The number of violent crimes in all seasons in each year slightly decreased over time (Supplementary Fig. S2 online). The rate of violent crime (count/10^5^ persons) ranged from 1274 to 3162 among the level-1 administrative areas (Supplementary Fig. S3 online). For the 201 level-2 administrative areas included in the analysis, the rate of violent crime (count/10^5^ persons) ranged from 490 to 5904 (mean = 1705, SD = 846). For sub-categories of violent crime, the number of incidences decreased about to 30% over the study years relative to the first year (i.e., 2016) for murder and robbery. The number of assaults slightly decreased by about 30% over time and the number of domestic violence crimes was similar across the study years (Supplementary Fig. S4 online).

**Figure 1:**
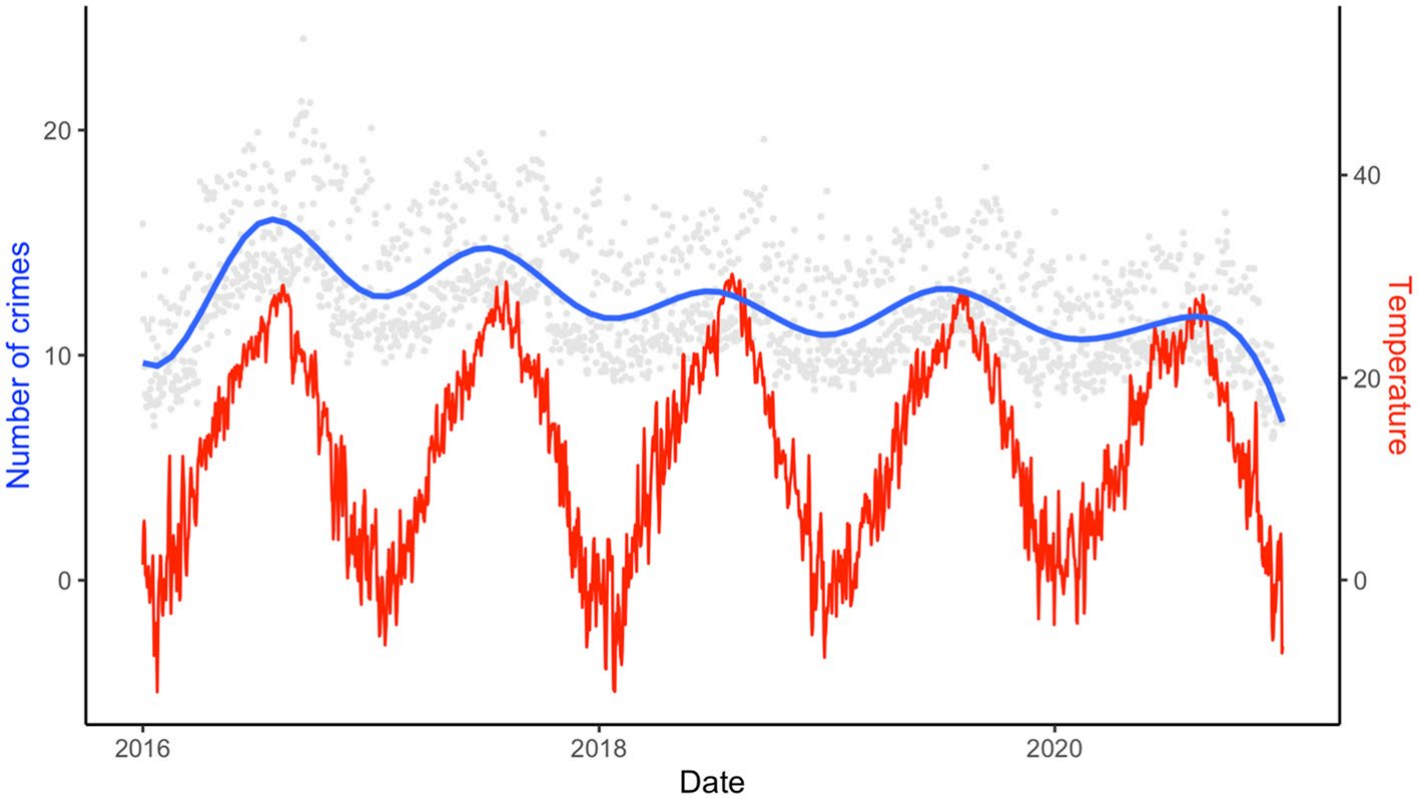
Time-series of the daily number of violent crimes and the average daily mean temperature (°C) across the study areas, from 1 January 2016 to 31 December 2020. The red line is for the daily mean temperature, and the blue smooth line is a LOESS curve of the daily number of violent crimes.

The number of violent crimes showed a seasonal pattern, with the highest numbers in hot seasons and the lowest numbers during cold seasons, and this pattern remained constant for all study years (Fig. 1).

**Figure 2:**
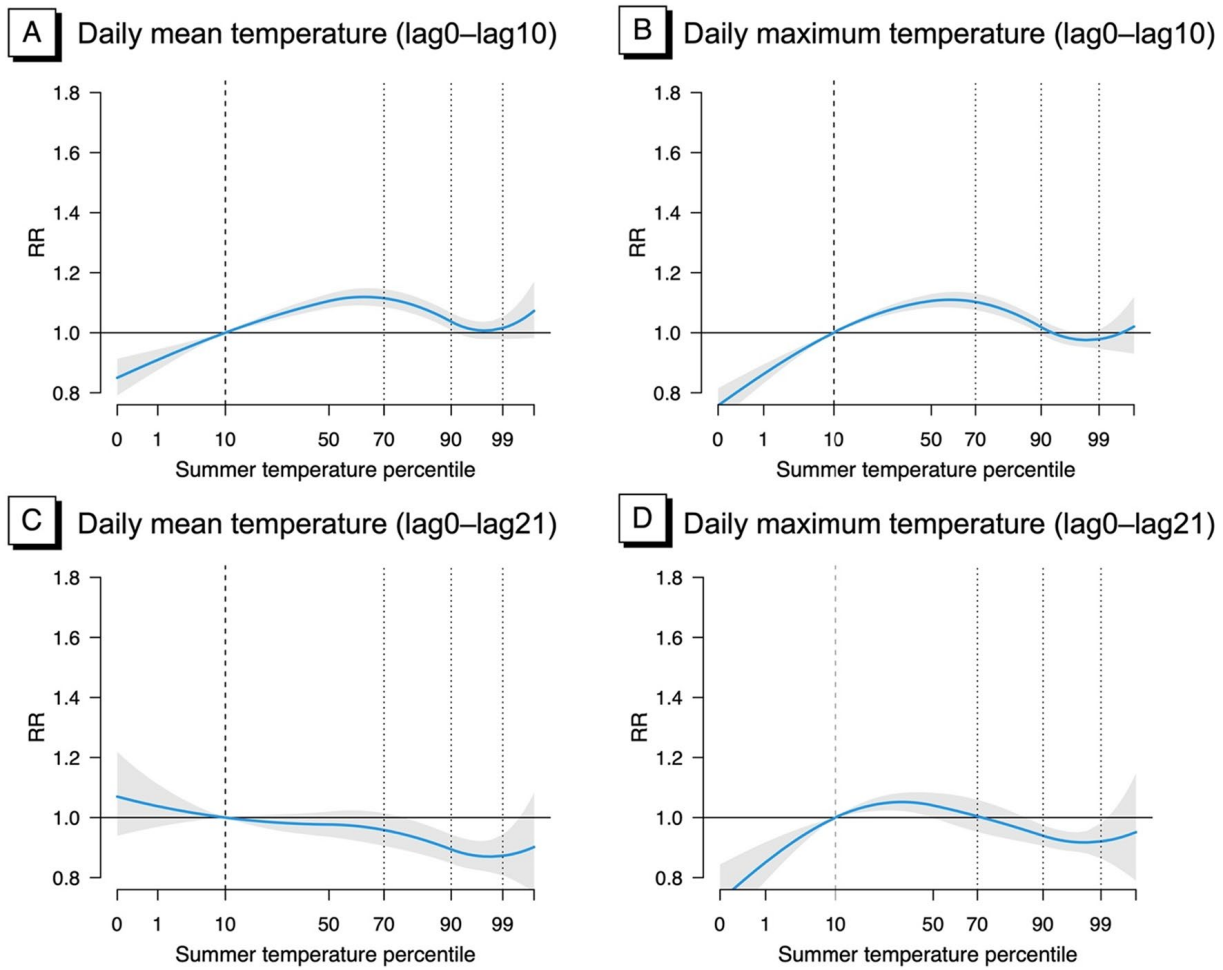
Overall cumulative exposure–response relationships for ambient temperature (lag0–lag10, lag0–lag21) during summer months and daily counts of violent crimes (2016–2020): (**A**) results for daily mean temperature (lag0–lag10), (**B**) results for daily maximum temperature (lag0–lag10), (**C**) results for daily mean temperature (lag0–lag21), and (**D**) results for daily maximum temperature (lag0–lag21) as temperature metrics.

The non-linear estimates of exposure–response relationships between lag0–lag10 temperature and violent crime are shown in Fig. 2. The pooled exposure–response relationships showed that the risks of violent crime increased with temperature with the highest risk around the 70th percentile of daily mean temperature. Risk decreased with rising temperatures for the temperature range roughly between the 70th and 97th percentiles. Above the 97th percentile for daily mean and maximum temperature a curve of increasing risks was found.

The pooled cumulative RRs of violent crime at the 70th, 90th, and 99th percentile values of daily mean temperature, compared to the reference percentile (i.e., 10th percentile) were 1.11 (95% CI 1.09, 1.15), 1.04 (95% CI 1.01, 1.07), and 1.02 (0.98, 1.06), respectively (Table 2). Significant associations were also found for the RRs at the 70th percentile value of daily maximum temperature compared to the reference percentile: 1.11 (1.08, 1.14).

**Table 2:** Pooled relative ratios (95% CI) of violent crime at the 70th, 90th, and 99th percentiles compared to the reference percentile (10th percentile) of daily mean temperature during lag0–10 days in summer months in South Korea.

	Daily mean temperature			Daily maximum temperature		
	RR: 70th vs. 10th	RR: 90th vs. 10th	RR: 99th vs. 10th	RR: 70th vs. 10th	RR: 90th vs. 10th	RR: 99th vs. 10th
Violent crime	1.11 (1.09, 1.15)	1.04 (1.01, 1.07)	1.02 (0.98, 1.06)	1.10 (1.08, 1.13)	1.02 (0.99, 1.04)	0.98 (0.95, 1.02)
Assault	1.12 (1.08, 1.15)	1.03 (1.00, 1.07)	1.02 (0.98, 1.07)	1.09 (1.06, 1.12)	1.01 (0.98, 1.04)	0.97 (0.93, 1.01)
Domestic violence	1.12 (1.05, 1.19)	1.06 (1.00, 1.12)	1.02 (0.94, 1.11)	1.11 (1.06, 1.16)	1.03 (0.99, 1.08)	1.00 (0.94, 1.07)

The models were adjusted for the season, calendar time, daily mean dew point temperature, national holidays, and day of the week.

The pooled cumulative RRs were estimated for sub-types of violent crime including assault and domestic violence (Table 2). The estimated RRs and 95% CIs of domestic violence at the 70th temperature percentiles of daily mean temperature compared to the reference temperature of the 10th percentile (1.12, 95% CI 1.05, 1.19) were similar to the estimated RRs of assault (1.12, 95% CI 1.08, 1.15) or violent crime overall (1.11, 95% CI 1.09, 1.15). Similar patterns were found for the results of daily maximum temperature (Table 2).

**Figure 3:**
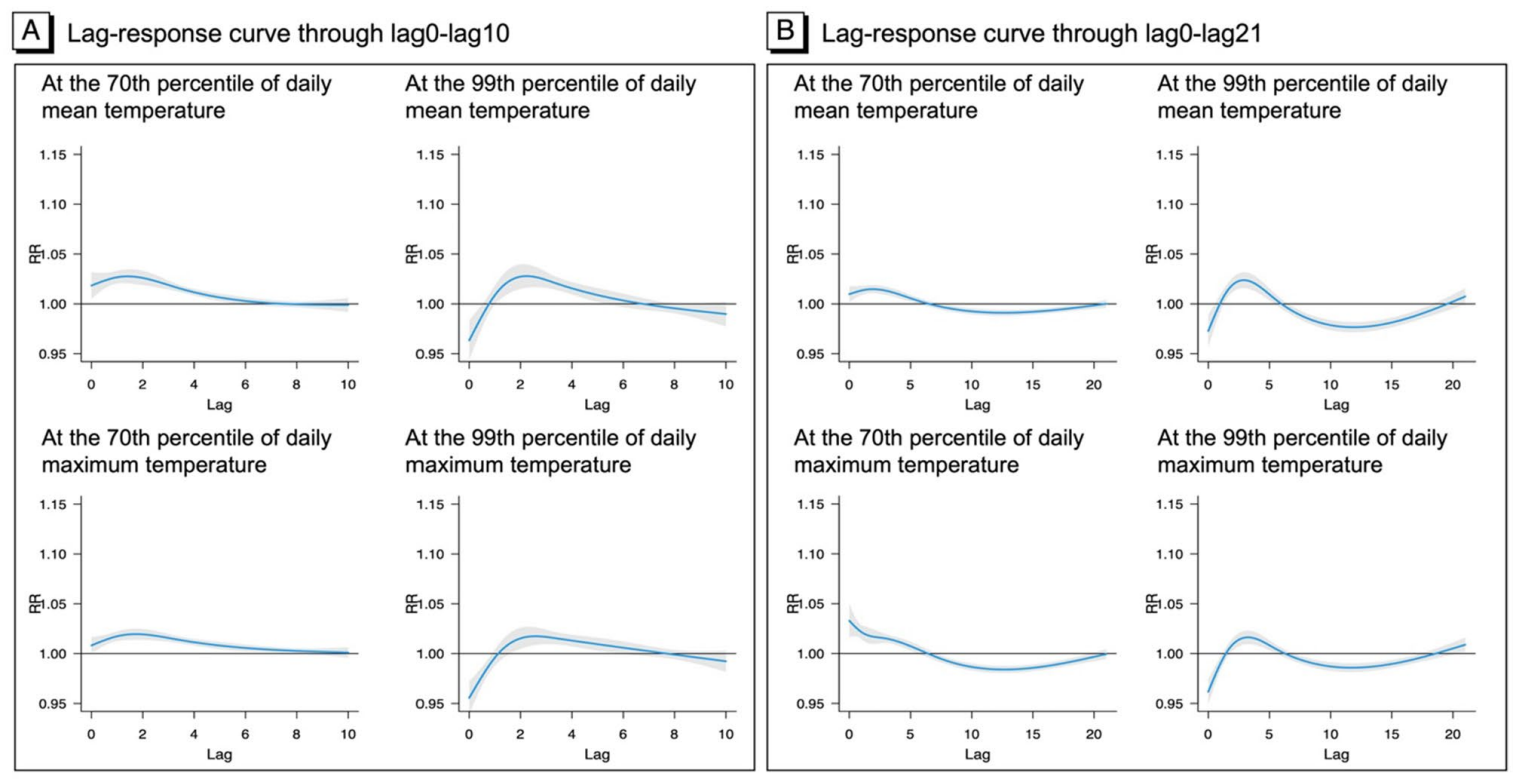
Lag-response curves between temperature and counts of violent crime predicted for the 70th and 99th summer percentile, with 95% CI.

The pooled percentile-specific lag-response relationships at the 70th and 99th summer temperature percentiles of daily mean and maximum temperatures showed lagged effects of short-term exposure to high temperature on violent crime (Fig. 3). The results are shown separately for exposure to lag0–lag10 temperature and lag0–lag21 temperature. For lag0–lag10 temperature, the RRs were the highest around lag 2 days and gradually decreased reaching an RR of 1 around 8–9 lag days. The lag-response curves based on lag0–lag21 showed negative RRs beyond lag6 due to displacement. As a result, the pooled cumulative RRs of violent crime through lag0–lag21 at the 70th, 90th, and 99th temperature percentiles compared to the reference temperature (10th percentile temperature) (Table S3 online) were smaller than the estimated pooled RRs of violent crime from the DLNM for lag0–10 temperature.

The RR of heat waves was not significant for heat waves defined as daily maximum temperature ≥ 33 °C for 2 or more consecutive days (RR = 0.99, 95% CI 0.95, 1.14) or days with the daily maximum temperature ≥ 35 °C for 2 or more consecutive days (RR = 1.03, 95% CI 0.97, 1.09).

The sensitivity analysis limiting the study period to 2016–2019 (excluding the COVID-19 pandemic influences) showed escalated risks of violent crime compared to the results of the main analysis. The RR at the 70th, 90th, and 99th summer temperature percentiles of daily mean temperature, compared to the 10th percentile, was 1.17 (95% CI 1.13, 1.21), 1.08 (95% CI 1.04, 1.12), and 1.04 (95% CI 0.99, 1.09), respectively. The RRs of daily maximum temperature at 70th, 90th, and 99th summer temperature percentiles were 1.18 (95% CI 1.14, 1.23), 1.08 (95% CI 1.04, 1.13), and 1.06 (95% CI 1.01, 1.12), respectively.

In other sensitivity analysis, the time-series analysis was applied for the entire years of the study period (2016–2020), rather than the warm months only. The model of daily mean temperature estimated the RRs at the 70th, 90th, and 99th temperature percentiles (new percentiles based on the 12-month full data) as 1.29 (95% CI 1.25, 1.33), 1.30 (95% CI 1.25, 1.34), and 1.21 (95% CI 1.17, 1.26), respectively. The results at the 70th, 90th, and 99th temperature percentiles based on the daily maximum temperature were 1.25 (95% CI 1.22, 1.27), 1.25 (95% CI 1.22, 1.28), and 1.15 (95% CI 1.12, 1.18), respectively.

The results of cumulative RRs through lag0–lag5 (Table S4 online) slightly increased RRs compared to the main analysis, resulting RRs that were not statistically significant at hot and extremely hot conditions. Due to the higher risks for lag days closer to the current day (i.e., lag0), a significant RR was found at the 99th percentile of daily mean temperature (RR = 1.03, 95% CI 1.00, 1.06). The RR at the 90th percentile of daily maximum temperature showed significant risks (RR = 1.03, 95% CI 1.01, 1.05).

Exponentiated coefficients of each temperature bin by the PPLM with fixed-effects are shown in Table S5. For violent crime (sum of murder, robbery, assault, and domestic violence), a non-linear exposure–response association was found. The risks grew from the first temperature bin until the fifth interval and after this interval the risk decreased until the last bin. Significantly positive associations were found between temperature and counts of all violent crime in the fifth and sixth bins, which indicates similar results to the DLNMs. Significant estimated impacts of temperature on increases in crime were found in one or more bins higher than the third temperature bin for all sub-type of the examined crime.

Results of pooled RRs of violent crime were robust to the adjustment for the confounding effects of daily PM_2.5_ and O_3_ concentrations (Table S8 online).

## Discussion

Climate effects on aggression and crime may be less important than the effects from other determinants such as social conditions^13^. Nonetheless, such contributions are still public safety concerns if entire populations are exposed and represent understudied potential consequences of climate change.

Violent crimes have seasonal patterns with higher rates in warm seasons in general^4,13,18^. The seasonal pattern is more pronounced for violent crimes, especially such as assault and domestic violence, in comparison to non-violent crimes^13^. In the current study, the daily violent crime counts were the highest during the summer months over the study years, consistent with findings from previous studies in several US cities including Boston, St. Louis, and Philadelphia^4,32,33^. In addition, our study found significant increases in violent crimes associated with short-term (i.e., daily) high temperature during the summer months in South Korea.

The suggested shapes of non-linear curves from previous modern studies of aggression and daily temperature included U-shaped, J-shaped, and inverted-U shape non-linear curves^13^. An inverted-U shape or inverted-J shape suggests an increase in violence as temperature rises, but only up to a specific temperature and reductions in violence for further increases in temperature^18^. Such patterns have been found in studies conducted in Dallas, TX and Philadelphia, PA, USA^32,54^. Laboratory studies suggest that although aggressive behaviors increase in relation to increases in temperature, further increases in temperature beyond a peak aggression point may decrease aggression^55^. When the predominant behavior tendency is escape, increased temperature can lead to increased actions of escape and decreased aggression; that is, the “negative affect escape model” argues that the negative affect of high temperature can lead to escape behaviors instead of aggressive behaviors^56^. Our results from the DLNMs showed that the relationships between daily temperature and violent crime counts were close to an inverted-U shape at the intermediate temperature (e.g., temperature < 90th percentile). For the extremely hot temperature ranges (e.g., temperature > 90th percentile), the relationship curve increased showing a U-shaped increase again. Our sensitivity analysis using a different analytic method based on the PPML regression found similar non-linear associations between average temperature during lag0–10 days and violent crime. The exposure–response relationship curves in our study differ from the findings from previous studies, possibly because our study focused on the daily variation in temperature during summer months, whereas most previous studies focused on the monthly or yearly variations in temperature. Also, the earlier studies were based on other study locations and populations, often on a single city. A study for Dallas, TX, USA showed an inverted U-shaped curve between temperature and violence for which the relationship became negative at around 32 °C^54^. An S-shaped curve with sharp peaks at low or high temperatures was also identified in some regions in previous studies^57^. In contrast, a study of Boston, MA, USA demonstrated a non-linear curve with a temperature cut-off around 25–27 Degrees of Heat Index where the risk of violent crimes started to increase stiffly^33^.

Identifying the underlying reasons for the shape of relationship between temperature and violent crime warrants further research. Acclimatization may play a role in this relationship. The exposure–response relationship curves shown in our study may imply the effects of high temperature through various mediated pathways^20^. For example, both the heat-aggression hypothesis and the negative affect escape model may be supportive of the mechanisms between high temperature and violent crimes in our study populations. The consistent increase in violent crime associated with a wide range of temperatures at which humans feel comfortable (e.g., temperature < 70th percentile) in this study could be because of the higher frequency of social encounters in moderate and warm weathers^58^. The Korean population may have adapted to the days with hot temperatures to cope with heat stress but not to the days with extremely hot temperatures. Activity patterns differ by level of temperature, and people may stay indoors at certain temperatures (e.g., above the 90th percentile) and use cooling measures (e.g., air conditioning), whereas extremely hot days may substantially trigger aggression and violence. Additional research on this non-linear association is needed. Despite the diversity in the shape of exposure–response relationships among quantitative studies (e.g., quadratic, linear, etc.), the evidence is robust for the increases in violent crime associated with high temperature across the studies^57^. The overarching associations between heat and violent crimes in this study are also consistent with previous studies. Several recent USA studies have examined the relationship between short-term temperature increase and violence in specific cities including St. Louis^4^, Chicago^58^, Boston^33^, and Philadelphia^32^. For example, extremely hot days (e.g., 27 °C < Heat Index) compared to very hot days (24 < Heat Index ≤ 27 °C) have about 1.31% higher rate (9% CI 0.18, 2.41) for assaults in a study conducted in Boston, MA, USA^33^. The rate of violent crimes was 9% higher when the daily mean Heat Index is at the 99th percentile (e.g., 31.6 °C) compared to the days with the daily mean Heat Index at the median (e.g., 13.6 °C) in Philadelphia^32^. Some non-US studies also suggested positive associations between high temperature and violence. A study focusing on 46 prefectures out of 47 in Japan found constantly increasing numbers of ambulance dispatches for assault as the temperature increases throughout all seasons^35^.

Our analysis examined the risks of violent crime for varying lagged effects of heat (e.g., lag0–lag10 and lag0–lag21). It is well established that the lagged effects of heat on various health outcomes (e.g., mortality) persist from the same day to 30 days after the exposure^36^. Some studies have suggested that the observed effects of heat on health outcomes may be partially explained by displacement, the phenomenon in which the exposure (e.g., heat) impacts already frail individuals whose health outcome (e.g., death) may have only been brought forward by a few days. If displacement occurs, an increase in mortality related to an exposure would be followed by a decrease in mortality to levels lower than expected mortality. Therefore, the displacement effect is an important consideration in accurate estimating the cumulative risks of outcomes. Many studies of heat and mortality have applied a 21-day lag in their model to examine how the displacement effect may affect estimated health impacts for a longer period than the first few days after an exposure. Potential displacement effects have been considered less among modern studies using crime statistics and heat exposure data. In our two models considering lag0–lag10 and lag0–lag21 exposures, the lag days yielding RRs less than 1 were similar (around lag7 or lag8). The estimated cumulative RRs of violent crime due to the lag0–lag21 heat exposure were smaller than the estimated RRs for the lag0–lag10 exposure as the model of lag0–lag21 exposures calculated the net effects including more lagged effects with RR < 1 over a longer period (i.e., 21 days). There is a lack of scientific consensus on the appropriate length of lagged effects that should be considered in statistical models for heat and violent crime, and further research is needed. Scientific evidence is needed regarding whether heat effects on violence and crime can be displaced by a year or longer^59^. Furthermore, knowledge gaps remain regarding how displacement effects may vary regionally within or between countries, warranting further research.

Violent crime measures substantially differ among the studies. Different definitions and categories of violent crime are included in the analysis. The words and categorizations for violent crimes can be also different by culture, country, and language. In the study for Boston, MA, USA, the daily violent crime counts were the aggregated numbers of aggravated assaults, simple assaults, crimes involving weapons, homicides, kidnappings, manslaughters, murders, escapes, runaways, truancies, and vandalism^33^. The study for Philadelphia, PA, USA defined violent crimes as robbery (with and without firearms), assaults (with and without firearms), homicides, and rapes^32^. The study for St. Louis, MO, USA included homicides, rapes, aggravated assaults, and robbery as violent crimes in their analysis^4^. Drug-trafficking organization killing rates were used to find associations between temperature and violence in a study in Mexico^60^. In our study, we defined violent crimes as murder, robbery, assault, and domestic violence, which were selected among the original categories in the raw dataset obtained from the Korean government. We note that data for some types of crimes, such as rape, were not available in the original dataset and may also be associated with high temperature. We also found that some studies had separate categories for simple assault and aggravated assault, but further categorizations for assault were not available in our dataset. The variety in the measures of violent crimes as a source of the heterogeneity of study findings should be considered in deriving the degree of evidence from future research in various communities and geographical regions. Given the heat-aggression theory^13^ and the study findings for the associations between high temperature and violent crime, some authors suggested that heat waves would likely increase interpersonal violence^61^. A study in Chicago, IL, USA suggested increases in non-domestic crime following the 5 days after heat wave days but not in domestic crime^58^. To the best of our knowledge, study efforts investigating the increased risks of violent crime during heat waves are far less common than the studies of the temperature-violent crime associations. Study results from the limited previous work on heat waves and violence appear to be inconclusive.

Studies of the temperature-violence relationships generally focused on the extreme forms of violent crimes such as aggravated assault and homicides^11,20^, and more research is warranted on other forms of interpersonal violence or non-violent crimes. A previous study from Minneapolis, MN, USA found a positive correlation between the number of calls for property crime and ambient temperature^62^. A study in Beijing, China showed that both violent and non-violent robbery rates increased as ambient heat stress increased at daily scales^31^. The impact of weather on crime could differ by types of crime. Our results based on the DLNM approach for incidence risks for each subtype of crime showed that heat was associated significant increases in burglary, robbery, assault, domestic violence, juvenile crime, misdemeanor, and traffic offense (Table S6 online). Our PPML regressions using six temperature bins fitting non-linear exposure–response functions also found significant estimated impacts of temperature on increased risks of these subtypes of crime especially above the area-specific 50th percentile of temperature (Table S5 online). Some forms of non-violent crimes including misdemeanor, and traffic offense in the Korean crime dataset may be associated with high temperature via psychological and pathogenic mechanisms of aggression, agitation, and impulsivity. Further research should be performed on the relationships between weather and different types of crime relevant to behavioral changes and aggression.

Violence is a public safety threat that needs to be treated with prevention and control efforts^63^. Despite the overall demonstrated association between temperature and violence, preventive measures for violent crime associated with temperature are uncertain^11^. Our findings that violent crime is related to temperature suggest that lower rates of violent crime are a potential co-benefit of mitigation policies of climate change and should be incorporated into discussions and analyses of climate change impacts and policies.

Future studies are required to examine the disparities of the associations between temperature and violent crime by various contextual factors. Factors that might be directly or indirectly associated with crime include environment, biology, spatial location, and density in addition to social and demographic factors (e.g., poverty, family, demographics, economics)^1^. An integrative approach for identifying potential effect modifiers is warranted in future studies. Multi-country and multi-region studies may assist in a better understanding of the disparities of the heat-violence associations by contextual factors, socioeconomic factors, and governance. Future studies should examine these factors to assist in the prevention of crime and promoting public health. For instance, studies examining economic health burdens due to weather-related violent crime are warranted as recovery from violence is a part of the health cost^5^.

Furthermore, identifying behavioral and mental factors of crime is important for the causation of exposure and response in epidemiologic frameworks^1^. For instance, aggression has been characterized with lower serotonin levels in the brain for several decades^64^. A previous observational study conducted in Finland, 1996–2013 examined the neurobiological mechanisms of seasonal temperature and violence by analyzing associations between monthly temperature, violent crime rates, and platelet serotonin transporters (SERT) levels. The results found a stronger negative correlation between SERT density and one-month delayed ambient temperature in violent offenders than healthy individuals. SERT density was also negatively correlated with the monthly violent crime rate. Future studies and data should better examine causal pathways of biological, behavioral, and mental factors to assist in the prevention of crime and promoting public health. Also, we examined the short-term effects of high ambient temperature on violent crime counts for a 5 year period, which is too short to study the impacts of climate change on violent crime with the consideration of long-term trends in temperature. Future studies are required to understand the causal associations between climate change and violence using a longer timeframe of data and accounting for the many other contributors to violence^65^.

We conducted a sensitivity analysis for the year 2020 to examine if the heat-violence associations differ during the COVID-19 pandemic. The RRs of total violent crime escalated at the 9th and 99th summer temperature percentiles compared to the reference temperature percentile (10th percentile) (Table S7 online). Growing evidence showed the negative influence of the COVID-19 pandemic on the worldwide population’s psychological wellbeing^66^. There is also emerging literature suggesting fundamental changes in people’s behaviors including the likelihood of crime, in particular domestic violence, due to the COVID-19 pandemic, while there has been mixed evidence regarding the degree to which incidents of domestic violence have changed compared to the pre-pandemic period among relevant studies^67^. Our results for the impact of heat on violent crime in 2020 may be influenced by the complex influences of environmental exposure on populations’ mental health, but more research will be required to examine them.

This study was conducted using the official national crime statistics in the study population. Our study included large numbers of neighborhood-level crime data, temperature, and confounding factors (e.g., dew point temperature, air pollution) for the entire country, which was valid for the study question and had representativeness of the populations compared to previous studies involving small samples of crime for a single city. Secondly, this study provides results for the temperature effects on sub-categories of violent crime including assault and domestic violence. This approach is notable as it was suggested that study results are particularly scarce for less extreme forms of violent crime such as domestic violence^11^, warranting more research efforts on these outcomes.

This study also has limitations. This study was not able to examine biological, psychological, physiological, and neurological pathways by which high temperature causes increases in violent crimes. Even though most of the existing studies are largely observational and thereby unable to fully test the theories of mechanisms, this does not mean a lack of evidence of associations^57^. Second, we did not have data for the behaviors of individuals or groups (e.g., aggression) or further details to distinguish characteristics of the crime (e.g., degree of aggression) or time of day. This led to an uncertainty of the temperature at the precise time the violent crime was performed and at the time the aggressive motivation (e.g., intention, mood) was initiated^13^. Third, although the models in this study adjusted for long-term trends in the daily count of violent crimes, the models did not consider socioeconomic factors as potential confounders. Also, our models were not able to adjust for some relevant individual-level modifiers such as acclimatization^58^ due to the lack of available data for individuals. Lastly, there may be an under-reporting of crime influencing the descriptive statistics of the crime in this study, which may differ by type of crime. Geographical heterogeneity for the potential under-reporting bias may exist as well. We assumed that the internal validity of the police-recorded crime data within a given area remained constant over the study period and therefore reporting bias would not significantly affect the area-specific estimation of the exposure–response relationships. Last, we used data for a period of a few years (i.e., 5 years from 2016 to 2020) and the long-term temporal changes in temperature and the heat-violence relationships in the study region were not captured in our analysis. Future studies are needed to examine the heat effects on violent crime over longer periods (e.g., decades) using historical and future projection data to better aid understanding of the risk of violent crime by climate change.

Heat stress and high temperature may relate to behavioral changes that can lead to violent crime through various mechanisms. However, study results are far limited for the associations between high temperature during the warm and hot seasons at daily scales and violent crimes at a population level. In summary, this study provided empirical evidence for the positive associations between daily temperature during summer months and counts of violent crimes in the Korean population. The estimated exposure–response relationships indicate increases in violent crimes with higher temperatures, within the temperate ranges of summer temperatures. This suggests the need for further understanding of the pathways through which climate-related variables can impact aggression and violence. Our findings thereby suggest the need for an extension of the climate change agenda to consider the impacts of climate and rising temperature on violent crime and promote public safety through mitigation and adaptation. Future studies are required for a better understanding of exposure–response relationships in different populations and for various types of crime and effective preventive measures for violent crimes triggered by high temperatures, and drivers of disparities in the temperature-violent crime associations.

### Supplementary Information

Supplementary Information.

## Data Availability

The data that support the findings of this study are available from the Smart Policing Big Data Platform (https://www.bigdata-policing.kr), but restrictions apply to the availability of these data, which were used under license for the current study, and so are not publicly available. Data are however available from the authors [corresponding author Seulkee Heo (seulkee.heo@yale.edu) and Michelle L. Bell (michelle.bell@yale.edu)] upon reasonable request and with permission of the Smart Policing Big Data Platform. Otherwise, the data can be directly purchased from the Smart Policing Big Data Platform on request.
