# Supplementary material for: A nationwide time-series analysis for short-term effects of ambient temperature on violent crime in South Korea
Source: Sci Rep. 2024 Feb 8;14:3210. doi: 10.1038/s41598-024-53547-6 (PMC10853231; doi:10.1038/s41598-024-53547-6)
Supplement: Supplementary file 1 — Supplementary Information. [file 41598_2024_53547_MOESM1_ESM.docx]

Supplementary Materials

A nationwide time-series analysis for short-term effects of ambient temperature on violent crime in South Korea

Seulkee Heo^1,*^, Hayon Michelle Choi^1^, Jong-Tae Lee^2^, Michelle L. Bell^1,2^

1 School of the Environment, Yale University, New Haven, CT, USA

2 Interdisciplinary Program in Precision Public Health, Department of Public Health Sciences, Graduate School of Korea University, Seoul, South Korea

Correspondence*: Seulkee Heo ([seulkee.heo@yale.edu](mailto:seulkee.heo@yale.edu)). 195 Prospect St, New Haven, CT 06511, USA


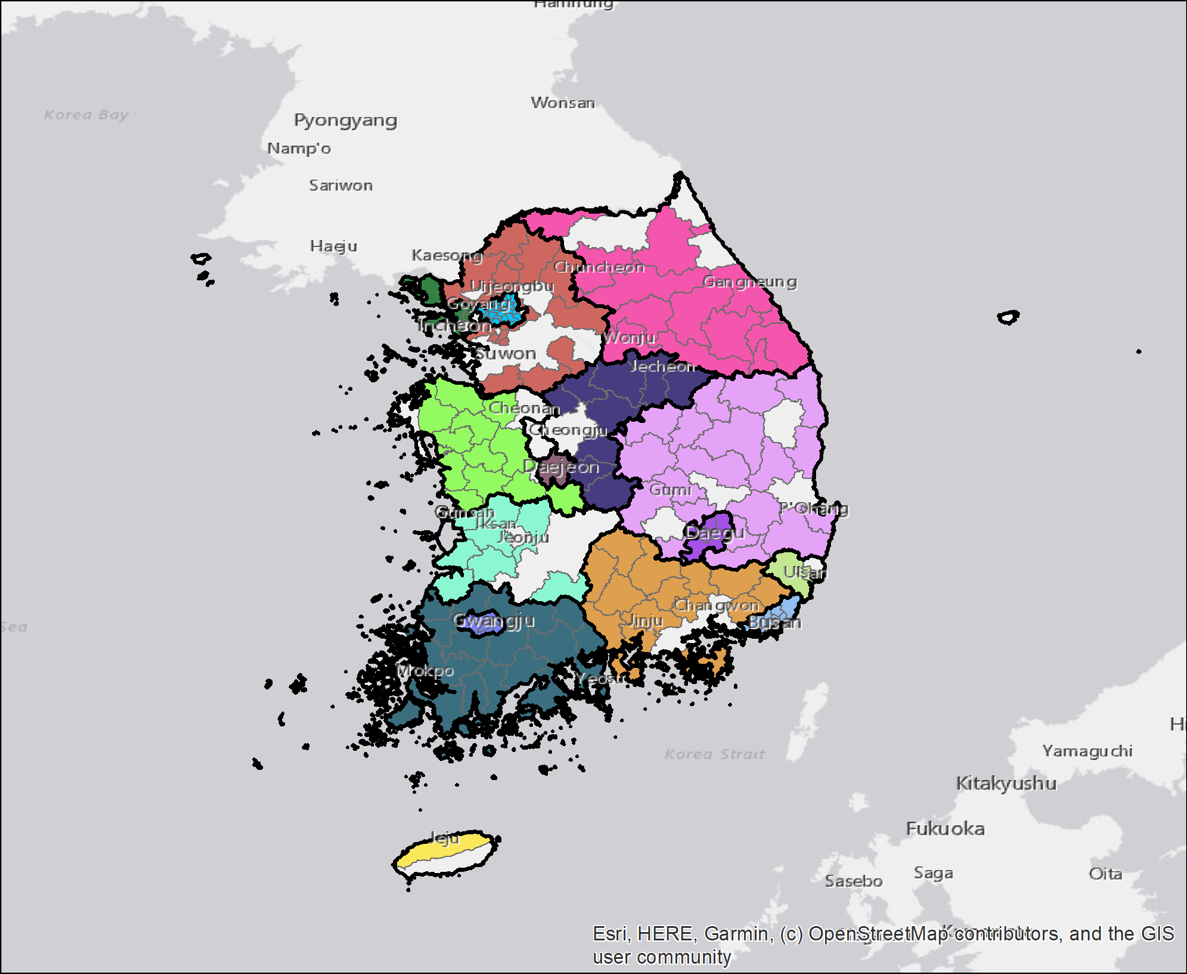


Figure S1. The level-2 administrative areas in South Korea. The 17 level-1 administrative containing 201 level-2 administrative areas in the analysis are shown in different colors. White areas in South Korea were excluded from the analysis due to the lack of crime data over the study years.


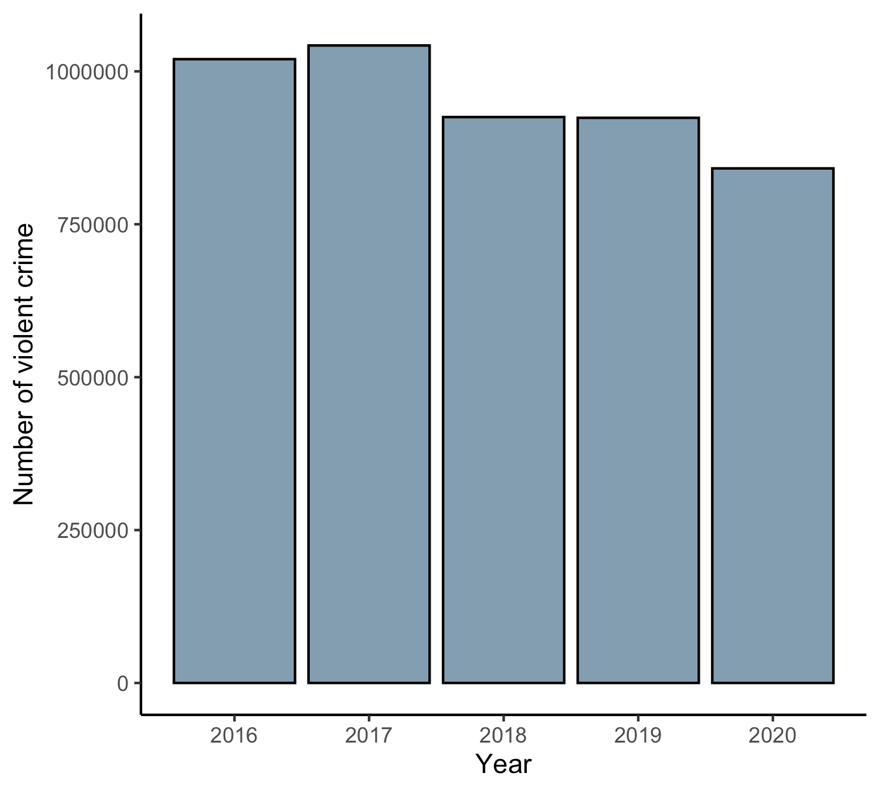


Figure S2. Counts of violent crime by year (2016-2020).


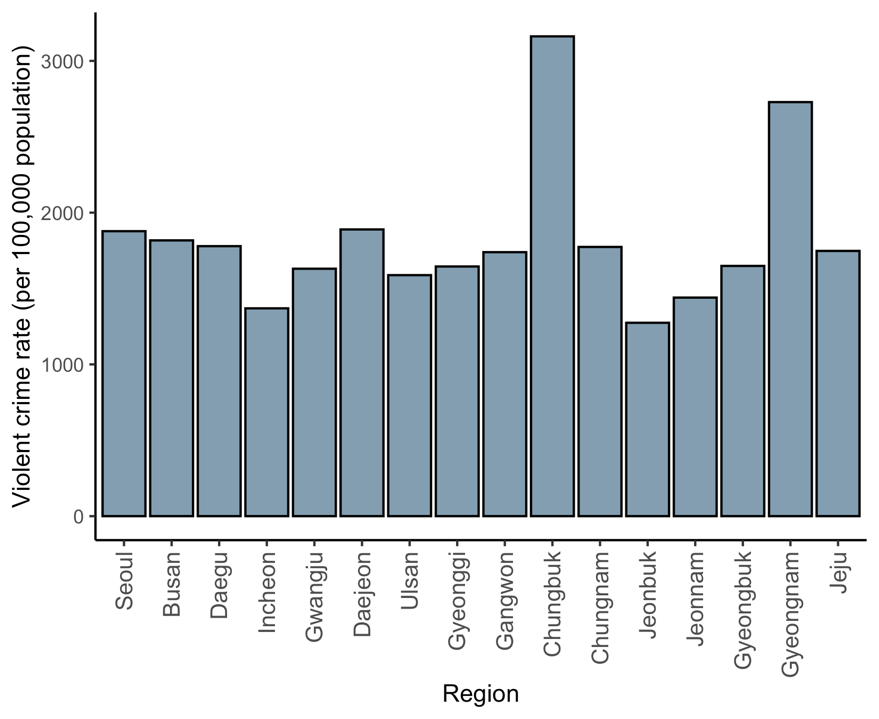


Figure S3. Average annual rate of violent crime in each level-1 administrative area in South Korea (2016-2020).


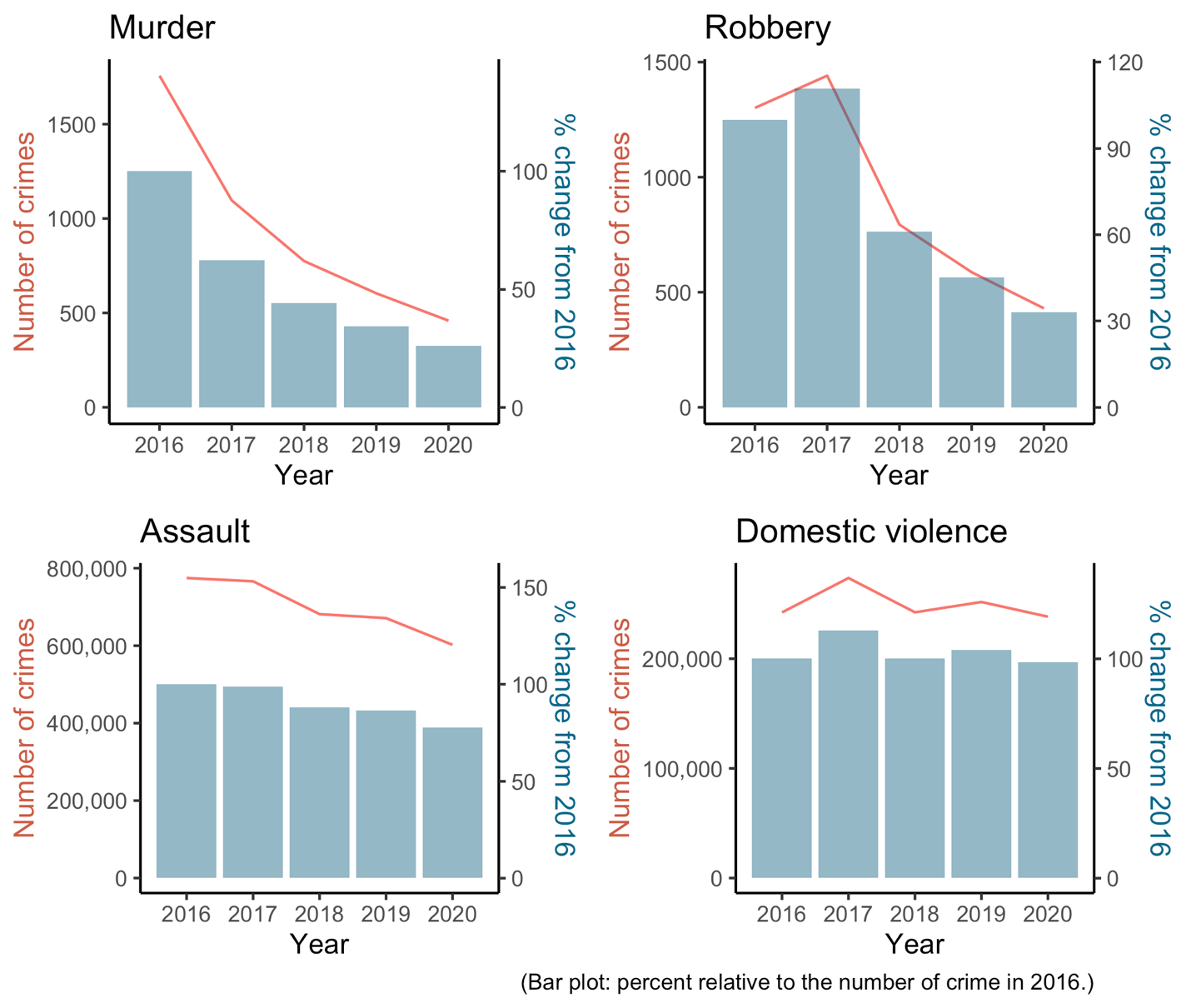


Figure S4. Counts of sub-categories of violent crime, stratified by year (2016-2020).

Note. The percentage of the y-axis on the right-hand side is the change in the number of crimes in each year relative to the number of crimes in 2016.

Table S1. Correlation matrix for daily air pollution and weather levels (January 2016 – December 2020).

|  | Daily mean temperature (°C) (mean, SD) | Daily maximum temperature (°C) (mean, SD) | Daily minimum temperature (°C) (mean, SD) | Daily mean dew point temperature (°C) (mean, SD) | Daily mean PM_2.5_ (µg/m^3^) (mean, SD) | Daily mean O_3_ (µg/m^3^) (ppm, SD) |
| --- | --- | --- | --- | --- | --- | --- |
| Daily mean temperature | 1.000 | 0.978 | 0.980 | 0.946 | -0.238 | 0.348 |
| Daily maximum temperature |  | 1.000 | 0.925 | 0.904 | -0.183 | 0.383 |
| Daily minimum temperature |  |  | 1.000 | 0.953 | -0.283 | 0.292 |
| Daily mean dew point temperature |  |  |  | 1.000 | -0.279 | 0.244 |
| Daily mean PM_2.5_ |  |  |  |  | 1.000 | 0.044 |
| Daily mean O_3_ |  |  |  |  |  | 1.000 |

Table S2. Correlation matrix for daily air pollution and weather levels in summer months (June – September), 2016-2020.

|  | Daily mean temperature (°C) (mean, SD) | Daily maximum temperature (°C) (mean, SD) | Daily minimum temperature (°C) (mean, SD) | Daily mean dew point temperature (°C) (mean, SD) | Daily mean PM_2.5_ (µg/m^3^) (mean, SD) | Daily mean O_3_ (µg/m^3^) (ppm, SD) |
| --- | --- | --- | --- | --- | --- | --- |
| Daily mean temperature | 1.000 | 0.887 | 0.893 | 0.722 | 0.127 | -0.050 |
| Daily maximum temperature |  | 1.000 | 0.615 | 0.466 | 0.242 | 0.116 |
| Daily minimum temperature |  |  | 1.000 | 0.845 | -0.012 | -0.212 |
| Daily mean dew point temperature |  |  |  | 1.000 | -0.060 | -0.274 |
| Daily mean PM_2.5_ |  |  |  |  | 1.000 | 0.432 |
| Daily mean O_3_ |  |  |  |  |  | 1.000 |

Table S3. Results of DLNMs for pooled cumulative relative ratios (95% CI) through lag0–lag21 of violent crime at the 70th, 90th, and 99th temperature percentiles compared to the reference temperature (10th percentile) in summer months in South Korea.

| Comparison | RR (95% CI) |
| --- | --- |
| Daily mean temperature |  |
| 70th vs. 10th | 0.96 (0.91, 1.01) |
| 90th vs. 10th | 0.89 (0.85, 0.94) |
| 99th vs. 10th | 0.87 (0.81, 0.94) |
| Daily maximum temperature |  |
| 70th vs. 10th | 1.00 (0.95, 1.06) |
| 90th vs. 10th | 0.94 (0.92, 0.97) |
| 99th vs. 10th | 0.92 (0.86, 0.98) |

Table S4. Results of DLNMs for pooled cumulative relative ratios (95% CI) through lag0–lag5 of violent crime at the 70th, 90th, and 99th temperature percentiles compared to the reference temperature (10th percentile) in summer months in South Korea.

| Comparison | RR (95% CI) |
| --- | --- |
| Daily mean temperature |  |
| 70th vs. 10th | 1.12 (1.10, 1.14) |
| 90th vs. 10th | 1.06 (1.04, 1.09) |
| 99th vs. 10th | 1.03 (1.00, 1.06) |
| Daily maximum temperature |  |
| 70th vs. 10th | 1.09 (1.07, 1.11) |
| 90th vs. 10th | 1.03 (1.01, 1.05) |
| 99th vs. 10th | 0.99 (0.96, 1.01) |

Table S5. Results of Poisson regressions with multiple fixed-effects: exponentiated coefficients and 95% CI of temperature bins (intervals).

| Outcome | Interval 1  (0-16th percentile) | Interval 2  (17-33rd percentile) | Interval 3  (34-50th percentile) | Interval 4  (51-67th percentile) | Interval 5  (68-84th percentile) | Interval 6  (85-99th percentile) |
| --- | --- | --- | --- | --- | --- | --- |
| All violent crime^a^ | 0.99 (0.98, 1.01) | 0.99 (0.98, 1.01) | 1.00 (0.99, 1.01) | 1.01 (1.00, 1.02) | 1.02 (1.01, 1.03) | 0.99 (0.98, 1.00) |
| Assault | 1.00 (0.98, 1.01) | 0.99 (0.98, 1.01) | 1.00 (0.99, 1.01) | 1.01 (1.00, 1.02) | 1.04 (1.02, 1.05) | 0.98 (0.96, 0.99) |
| Domestic violence | 0.98 (0.95, 1.00) | 1.00 (0.98, 1.02) | 0.98 (0.96, 1.00) | 1.01 (0.99, 1.02) | 1.00 (0.97, 1.02) | 1.03 (1.01, 1.05) |
| Burglary | 1.01 (0.99, 1.03) | 0.99 (0.97, 1.01) | 1.00 (0.98, 1.01) | 1.01 (0.99, 1.02) | 0.96 (0.95, 0.97) | 1.03 (1.01, 1.04) |
| Sexual crime | 0.96 (0.93, 0.99) | 0.98 (0.95, 1.01) | 1.01 (0.99, 1.04) | 1.03 (1.01, 1.06) | 0.99 (0.97, 1.02) | 0.96 (0.93, 0.99) |
| Public indecency | 1.03 (1.00, 1.06) | 0.99 (0.96, 1.02) | 1.00 (0.98, 1.02) | 1.03 (1.01, 1.05) | 1.00 (0.96, 1.04) | 0.99 (0.96, 1.02) |
| Juvenile crime | 0.98 (0.96, 1.01) | 1.01 (0.98, 1.04) | 0.99 (0.97, 1.02) | 1.08 (1.05, 1.10) | 1.08 (1.05, 1.11) | 0.94 (0.91, 0.97) |
| Misdemeanor | 0.98 (0.97, 0.99) | 1.00 (0.99, 1.01) | 0.99 (0.98, 1.00) | 1.00 (0.99, 1.01) | 1.00 (0.99, 1.01) | 1.01 (1.00, 1.02) |
| Traffic offense | 1.00 (0.99, 1.01) | 0.99 (0.98, 1.00) | 0.94 (0.98, 0.99) | 1.00 (0.99, 1.01) | 1.00 (0.99, 1.02) | 1.00 (0.99, 1.02) |
| “Other” crime | 1.00 (0.99, 1.01) | 1.00 (0.99, 1.01) | 1.00 (0.99, 1.01) | 1.01 (1.00, 1.02) | 1.01 (1.00, 1.02) | 1.00 (0.99, 1.01) |

Note. a: Sum of murder, robbery, assault, and domestic violence.

Table S6. Results of DLNMs for pooled cumulative relative risks for subtypes of crime (2016–2020) at 70th, 90th, and 99th temperature percentiles compared to the reference temperature (10th percentile).

| Outcome | Daily mean temperature | | | Daily maximum temperature | | |
| --- | --- | --- | --- | --- | --- | --- |
|  | RR: 70th vs. 10th | RR: 90th vs. 10th | RR: 99th vs. 10th | RR: 70th vs. 10th | RR: 90th vs. 10th | RR: 99th vs. 10th |
| Murder | 2.53 (0.40, 16.05) | 3.23 (0.43, 19.23) | 0.25 (0.04, 1.40) | 1.54 (0.40, 6.00) | 1.54 (0.63, 3.76) | 0.07 (0.01, 0.39) |
| Robbery | 2.33 (1.18, 4.62) | 1.77 (0.81, 3.88) | 0.31 (0.10, 1.00) | 2.13 (0.97, 4.67) | 1.95 (0.91, 4.22) | 0.27 (0.11, 0.66) |
| Burglary | 1.09 (1.04, 1.13) | 1.14 (1.07, 1.20) | 1.19 (1.13, 1.25) | 1.09 (1.04, 1.13) | 1.14 (1.07, 1.20) | 1.19 (1.13, 1.25) |
| Assault | 1.05 (1.02, 1.09) | 0.99 (0.95, 1.03) | 1.01 (0.96, 1.06) | 1.05 (1.02, 1.09) | 0.99 (0.95, 1.03) | 1.01 (0.96, 1.06) |
| Domestic violence | 1.03 (1.00, 1.07) | 1.00 (0.96, 1.05) | 0.97 (0.90, 1.05) | 1.03 (1.00, 1.07) | 1.00 (0.96, 1.05) | 0.97 (0.90, 1.05) |
| Sexual crime | 1.01 (0.93, 1.10) | 0.97 (0.92, 1.03) | 1.01 (0.90, 1.15) | 1.00 (0.92, 1.09) | 0.98 (0.91, 1.06) | 1.01 (0.90, 1.13) |
| Public indecency | 0.93 (0.86, 1.00) | 0.79 (0.72, 0.87) | 0.83 (0.75, 0.92) | 0.91 (0.86, 0.96) | 0.82 (0.76, 0.88) | 0.88 (0.81, 0.96) |
| Juvenile crime | 1.26 (1.18, 1.35) | 1.09 (1.05, 1.13) | 1.14 (1.07, 1.23) | 1.18 (1.10, 1.26) | 1.01 (0.94, 1.08) | 1.06 (0.98, 1.15) |
| Misdemeanor | 1.10 (1.07, 1.13) | 1.09 (1.06, 1.13) | 1.10 (1.06, 1.14) | 1.07 (1.04, 1.11) | 1.05 (1.02, 1.08) | 1.05 (1.02, 1.09) |
| Traffic offense | 1.03 (0.99, 1.07) | 1.03 (0.99, 1.06) | 1.01 (0.95, 1.07) | 1.00 (0.97, 1.03) | 1.02 (1.00, 1.04) | 0.98 (0.92, 1.05) |
| “Other” crime | 1.07 (1.05, 1.08) | 1.04 (1.01, 1.06) | 1.04 (1.00, 1.09) | 1.05 (1.03, 1.08) | 1.01 (0.99, 1.03) | 1.03 (0.99, 1.08) |

Note. a: Among the 13 subtypes of crime, two types of crime (fraud, voice phishing) were excluded from this analysis. The results in this table are based on the analyses using exposure and crime data aggregated for each level-1 administrative area.

Table S7. Results of DLNMs for pooled cumulative relative risks of violent crime in the year 2020 with the COVID-19 pandemic.

| Comparison | Daily mean temperature | Daily maximum temperature |
| --- | --- | --- |
| RR: 70th vs. 10th | 1.14 (1.00, 1.29) | 1.04 (0.93, 1.15) |
| RR: 90th vs. 10th | 1.25 (1.08, 1.46) | 1.12 (1.02, 1.22) |
| RR: 99th vs. 10th | 1.45 (1.21, 1.74) | 1.29 (1.13, 1.47) |

Table S8. Pooled relative ratios (95% CI) of violent crime at 70th, 90th, and 99th temperature percentiles compared to the reference temperature (10th percentile), with adjustments for air pollution (PM_2.5_, O_3_) in DLNMs.

|  | Daily mean temperature | | | Daily maximum temperature | | |
| --- | --- | --- | --- | --- | --- | --- |
|  | RR: 70th vs. 10th | RR: 90th vs. 10th | RR: 99th vs. 10th | RR: 70th vs. 10th | RR: 90th vs. 10th | RR: 99th vs. 10th |
| Violent crime | 1.11  (1.08, 1.14) | 1.03  (1.00, 1.06) | 1.02  (0.98, 1.06) | 1.10  (1.07, 1.13) | 1.01  (0.99, 1.04) | 0.99  (0.95, 1.02) |

Note. The models were also adjusted for the season, calendar time, daily mean dew point temperature, national holidays, and day of the week.
